# Thermoelectric Performance of n-Type Magnetic Element Doped Bi_2_S_3_

**DOI:** 10.1021/acsaem.2c00295

**Published:** 2022-03-01

**Authors:** Raphael Fortulan, Sima Aminorroaya Yamini, Chibuzor Nwanebu, Suwei Li, Takahiro Baba, Michael John Reece, Takao Mori

**Affiliations:** † Materials and Engineering Research Institute, Sheffield Hallam University, Sheffield S1 1WB, U.K.; ‡ Department of Engineering and Mathematics, Sheffield Hallam University, Sheffield S1 1WB, U.K.; § School of Engineering and Material Science, Queen Mary University of London, Mile End Road, London E1 4NS, U.K.; ∥ International Center for Materials Nanoarchitectonics (WPI-MANA), National Institute for Materials Science, Tsukuba 305-0044, Japan; ⊥ Graduate School of Pure and Applied Science, University of Tsukuba, Tsukuba 305-8577, Japan

**Keywords:** thermoelectric, power factor, magnetic element, bismuth sulfide, chromium

## Abstract

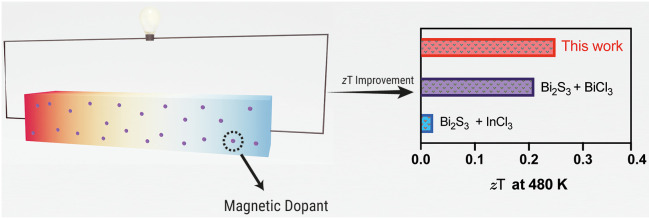

Thermoelectric technology offers great potential for converting waste heat into electrical energy and is an emission-free technique for solid-state cooling. Conventional high-performance thermoelectric materials such as Bi_2_Te_3_ and PbTe use rare or toxic elements. Sulfur is an inexpensive and nontoxic alternative to tellurium. However, achieving high efficiencies with Bi_2_S_3_ is challenging due to its high electrical resistivity that reduces its power factor. Here, we report Bi_2_S_3_ codoped with Cr and Cl to enhance its thermoelectric properties. An enhanced conductivity was achieved due to an increase in the carrier concentration by the substitution of S with Cl. High values of the Seebeck coefficients were obtained despite high carrier concentrations; this is attributed to an increase in the effective mass, resulting from the magnetic drag introduced by the magnetic Cr dopant. A peak power factor of 566 μW m^–1^ K^–2^ was obtained for a cast sample of Bi_2–*x*/3_Cr_*x*/3_S_3–*x*_Cl_*x*_ with *x* = 0.01 at 320 K, as high as the highest values reported in the literature for sintered samples. These results support the success of codoping thermoelectric materials with isovalent magnetic and carrier concentration tuning elements to enhance the thermoelectric properties of eco-friendly materials.

## Introduction

Solid-state-based thermoelectric (TE) materials can directly and reversibly convert heat into electricity. The efficiency of thermoelectric materials is given by the figure of merit, *zT* = (*S*^2^*T*)/ρκ_total_, where *S* is the Seebeck coefficient, *T* is the absolute temperature, ρ is the electrical resistivity, and κ_total_ is the thermal conductivity.

To increase *zT*, one needs to increase the power factor (*S*^2^/ρ) and/or decrease κ_total_. One of the most successful approaches to improve the figure of merit is reducing the lattice thermal conductivity, and over the years, various phonon engineering approaches have been used to enhance phonon scattering and decrease κ_L_ by taking advantage of nanoprecipitates,^1,2^ alloying elements,^3,4^ nanostructured grain boundaries,^5,6^ and ionized impurities.^7,8^

A series of band structure engineering approaches have also been employed to improve the power factor of TE materials.^9−11^ Strategies such as quantum confinement,^12^ modulation doping,^13−15^ and energy filtering^16,17^ are being actively pursued.

Magnetic interactions have been proposed as a strategy to enhance the Seebeck coefficient in thermoelectric materials such as Bi_2_Te_3_.^18−23^ Charge carriers interact with the local magnetic moments, effectively dragging the carriers, which results in an increased charge carrier effective mass, an increased Seebeck coefficient, and a decreased carrier mobility (μ). Overall, this has resulted in an increased power factor.^18−24^

Tellurium-based thermoelectric materials such as Bi_2_Te_3_ have been employed as power generators/refrigerators in lower-temperature applications (<500 K). However, tellurium is expensive and rare and can hinder the movement toward the mass adoption of TE generators. Sulfur, another element from group IV, is an inexpensive, nontoxic, and sustainable alternative. Bismuth sulfide (Bi_2_S_3_), in particular, has low thermal conductivity and a large Seebeck coefficient.^25,26^ However, its high resistivity results in a low *zT*.^27^ Several dopants have been used to optimize the electronic transport properties of Bi_2_S_3_, including CuBr_2_,^28^ Sb,^29^ Cu,^30^ Ag,^31^ I,^32^ Cl,^33^ Se,^33,34^ InCl_3_,^35^ BiCl_3_,^36^ and NbCl_5_.^37^ A lower thermal conductivity was also obtained in Bi_2_S_3_ by nanostructuring.^30,38−40^

The thermoelectric efficiency of pristine Bi_2_S_3_ was also increased to 0.11 from 0.09 at 623 K by texturing through hot forging and introducing sulfur vacancies.^41^ PbBr_2_ doping of bulk Bi_2_S_3_ has significantly improved its electrical conductivity by modulation doping and reduced the lattice thermal conductivity by introducing nanoprecipitates, resulting in a peak *zT* value of 0.8 at 673 K.^42^

It has been widely shown that the charge density is increased when halogen group elements (Cl, Br, and I) are doped at the sulfur sites.^28,32,33^ Here, we doped bismuth sulfide with chromium chloride (CrCl_3_) to obtain samples of Bi_2–*x*/3_Cr_*x*/3_S_3–*x*_Cl_*x*_ (*x* = 0.00, 0.005, 0.01, 0.015, 0.02). Doping with chlorine increases the number of free carriers in the material, leading to a reduction in the electrical resistivity, while the magnetic effect of chromium resulted in an increase in the carrier effective mass and, consequently, in the Seebeck coefficient.

## Experimental Section

### Sample Fabrication

Ultrahigh-purity bismuth pieces (99.999%, Sigma-Aldrich), sulfur pieces (99.9995%, Alfa Aesar Puratronic), and chromium chloride powder (99.99%, Sigma-Aldrich) were mixed stoichiometrically to obtain Bi_2–*x*/3_Cr_*x*/3_S_3–*x*_Cl_*x*_ (*x* = 0.00, 0.005, 0.01, 0.015, 0.02) in vacuum-sealed quartz ampules, prepared in an inert-atmosphere glovebox. The tubes were heated in a tube furnace to 1000 °C. After being quenched in cold water, the samples were annealed at 450 °C for 2 days.

The cylindrical ingot samples of 10 mm diameter were then cut into disk shapes of 10 mm diameter and ∼1.5 mm thickness for Hall effect measurements and bars of 2 × 2 × 10 mm^3^ for electrical property measurements. The electrical resistivity and Seebeck coefficient were measured simultaneously under 0.1 bar of helium from room temperature to 483 K using an LSR-3 Linseis unit. Hall effect measurements were performed with an Ecopia HMS-3000 Hall Measurement System at room temperature. The density of the samples was determined from the bar-shaped samples using their dimensions and masses. All samples were then manually ground to fine powders by using an agate mortar and pestle. Three samples with *x* = 0, 0.005, and 0.01 were sintered in a 10 mm diameter graphite die under an axial pressure of 63 MPa at 723 K for 5 min under vacuum; the sample with *x* = 0.01 broke during sintering. To avoid this, the sintering temperature was reduced to 623 K for the samples with compositions of *x* = 0.015, 0.02. The measured densities of all samples are presented in Tables S1 and S2 in the Supporting Information.

### Material Characterization

**Figure 1:**
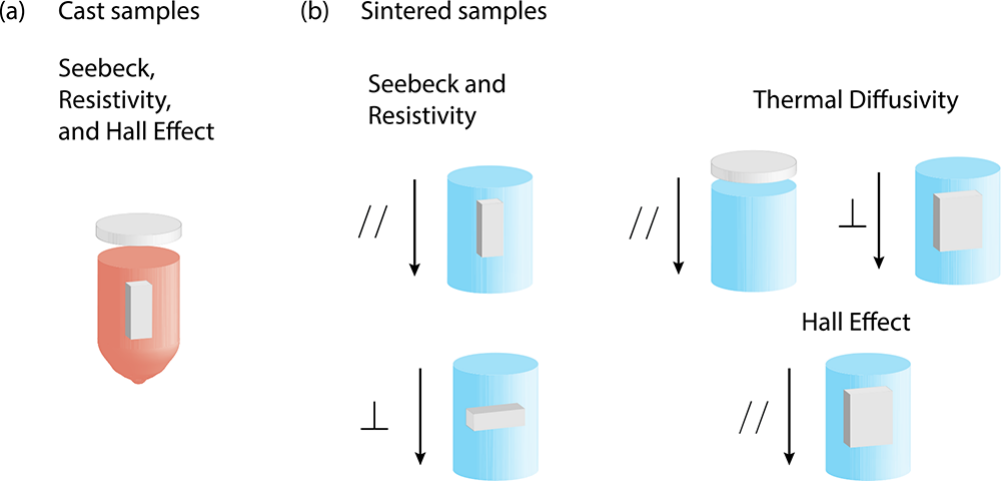
Measuring directions and sample shapes of the (a) cast samples and (b) sintered samples.

To investigate the electrical and thermal transport properties parallel and perpendicular to the sintering direction, the sintered samples were cut and polished into disks (10 mm diameter and ∼1.5 mm thickness, perpendicular to the pressing direction) and cuboids of 8 × 8 × 2 mm^3^ parallel to the pressing direction for Hall effect and thermal diffusivity measurements and bars of 2 × 2 × 10 mm^3^ (parallel and perpendicular to the pressing direction) for electrical property measurements. The total thermal conductivity (κ_total_) was calculated from the thermal diffusivity (*D*), heat capacity (*C*_p_) and density (ρ): κ_total_ = *DC*_p_ρ. The temperature-dependent thermal diffusivity *D* was measured on disk-shaped samples by a laser flash diffusivity method using a Netzsch LFA-467 Hyperflash instrument. The temperature-dependent heat capacity was derived using a standard sample (Pyroceram-9060). The directions of measurement and sample shapes are illustrated in Figure 1. X-ray powder diffraction analysis was performed with a PANalytical X’Pert PRO instrument, using Cu Kα1 radiation (λ = 1.54059 Å) to identify the crystal structure of each sample. Rietveld refinement was performed using GSAS-II^43^ to obtain the lattice parameters for all samples.

### Electronic Structure Calculation

Density functional theory (DFT) calculations were employed to qualitatively study the electronic band structure of the doped sample. The Perdew–Burke–Ernzerhof (PBE) and generalized gradient approximation (GGA) exchange-correlation functionals were used^44^ with the Quantum Espresso package.^45^ A Monkhorst–Pack procedure was used to generate 12 × 12 × 12 *k*-points for the Brillouin zone.^46^ The plane wave/pseudopotential approach was employed, with a kinetic energy cutoff of 45 Ry for the wave functions and 360 Ry for the electron density. Spin polarization was considered for the materials doped with Cr.

## Results and Discussion

### Materials Characteristics

**Figure 2:**
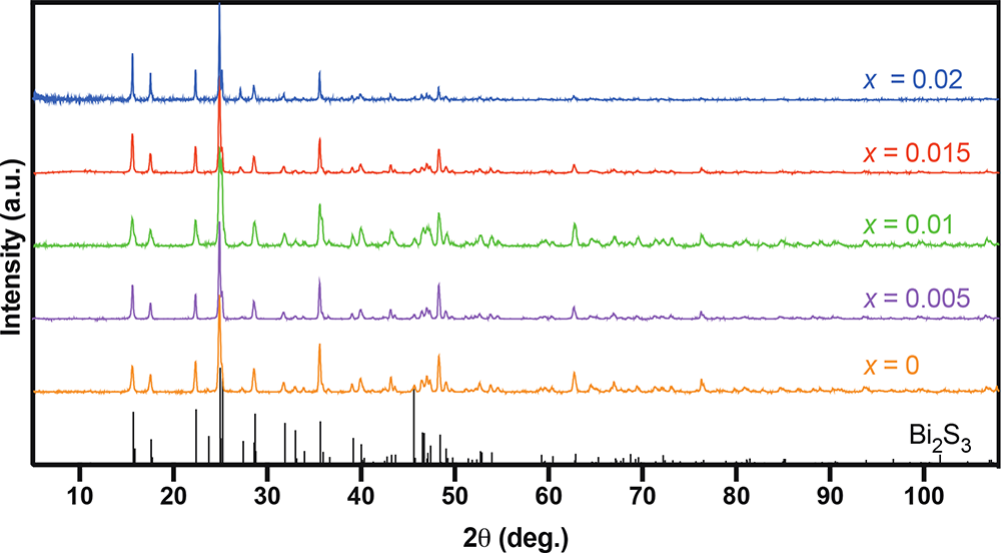
Powder XRD patterns of Bi_2–*x*/3_Cr_*x*/3_S_3–*x*_Cl_*x*_ (*x* = 0.00, 0.005, 0.01, 0.015, 0.02) samples in the range of 5–108°.

Figure 2 shows the XRD patterns of samples Bi_2–*x*/3_Cr_*x*/3_S_3–*x*_Cl_*x*_ (*x* = 0.00, 0.005, 0.01, 0.015, 0.02). All patterns confirm the presence of a single-phase Bi_2_S_3_, orthorhombic crystal structure with space group *Pnma*. The lattice parameters of all the samples were determined by the Rietveld refinement of the XRD patterns (Table S3 in the Supporting Information). No variation of the lattice parameters was detected, due to the comparable ionic radii of S^2–^ (1.84 Å) and Cl^–^ (1.81 Å).^47^ Although there is a difference in the ionic radii of Bi^3+^ (1.03 Å) and Cr^3+^ (0.615 Å),^47^ the amount of chromium introduced to the Bi_2_S_3_ is one-third of the chlorine atomic ratio, and therefore no noticeable difference was detected in the lattice parameters.

The lattice parameter values are consistent with the values reported in the literature (*a* = 11.269 Å, *b* = 3.972 Å, and *c* = 11.129 Å).^48^

The intensity of the {111} plane peaks for the *x* = 0.015 sample was higher than those for the other samples. This might be attributed to the preferred orientation, caused by nonuniform hand milling of the samples used for the XRD analysis.

**Figure 3:**
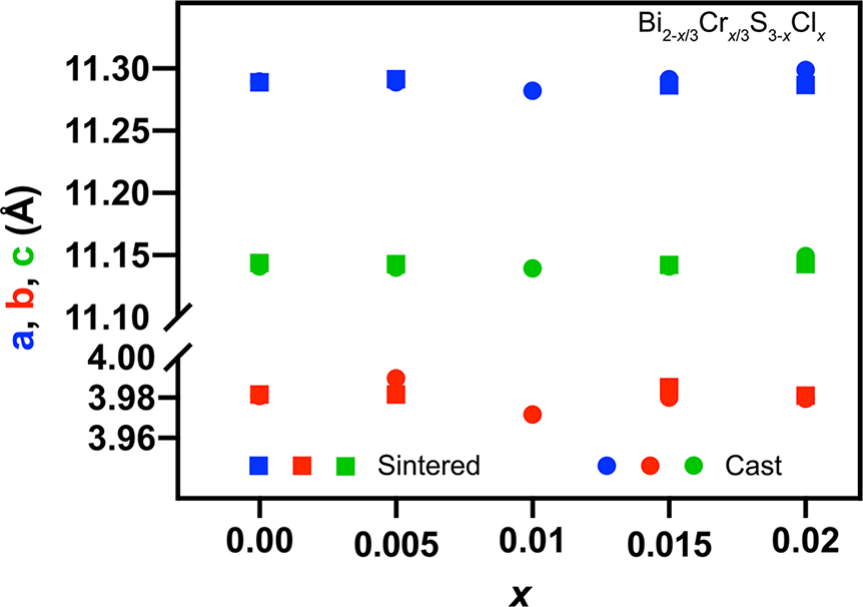
Rietveld refined lattice parameters of Bi_2–*x*/3_Cr_*x*/3_S_3–*x*_Cl_*x*_ (*x* = 0.00, 0.005, 0.01, 0.015, 0.02) samples as a function of the dopant concentration.

An XRD analysis was also performed on the sintered samples (Figure S1 in the Supporting Information), and the lattice parameters were calculated by a Rietveld refinement (Table S4 in the Supporting Information). The lattice parameter values of Bi_2–*x*/3_Cr_*x*/3_S_3–*x*_Cl_*x*_ (*x* = 0.00, 0.005, 0.01, 0.015, 0.02) samples versus the dopant concentration (*x*) of cast and sintered samples are shown in Figure 3.

**Figure 4:**
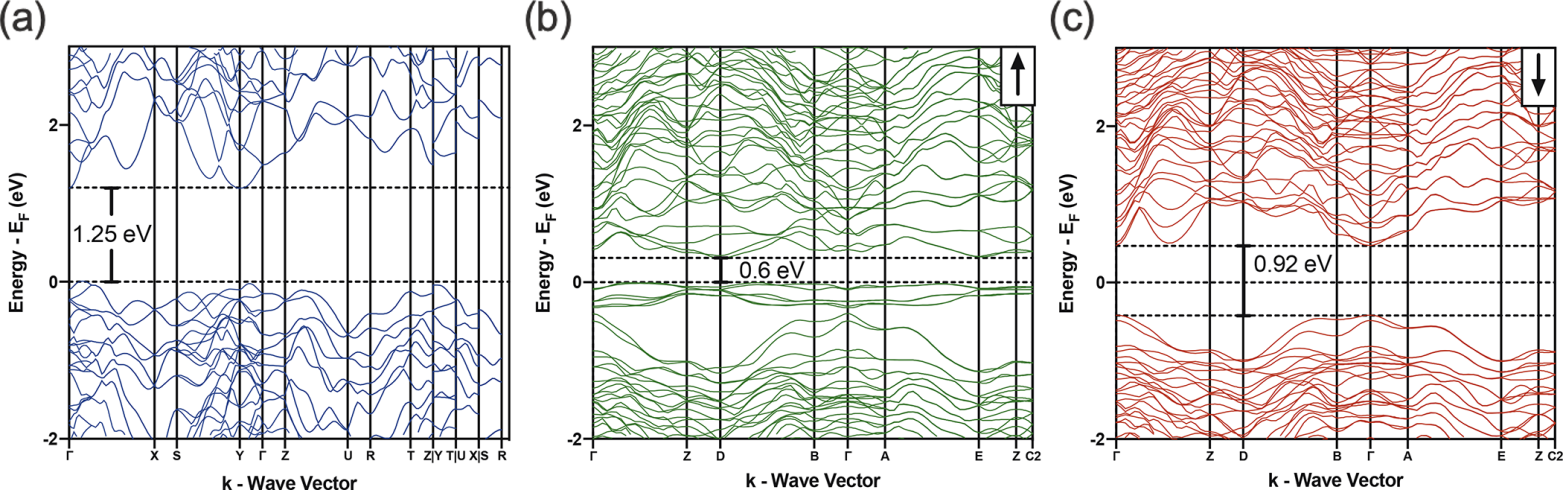
Electronic band structure of (a) Bi_2_S_3_, (b) Bi_23_Cr_1_S_33_Cl_3_ spin-up (↑) state, and (c) Bi_23_Cr_1_S_33_Cl_3_ spin-down (↓) state.

To understand the effect of dopants on the electronic band structure of Bi_2_S_3_, the band structures of Bi_2_S_3_ and the doped sample Bi_23_Cr_1_S_33_Cl_3_, for spin-up and -down states, were calculated (Figure 4a–c, respectively). The calculated band gap of the pristine material is ∼1.25 eV, which is in good agreement with the reported experimental values of ∼1.3 eV.^35,49,50^ Both spin-up and spin-down states showed reduced values of ∼0.6 and ∼0.92 eV, respectively. The reduction in the band gap for the spin-up state was due to the presence of an additional impurity band. It is worth noting that the numerical results, presented in this calculation, should only be discussed qualitatively due to the rather high concentration of the dopant. The effective masses of electrons were calculated for both heavy and light bands in the spin-up (D point) and spin-down (Γ point) states of the electronic band structures, using the parabolic band approximation for the band extrema. The results are shown in Figure S2 in the Supporting Information. The electrons of both heavy and light bands show similar values of effective mass (*m*_heavy_* ≈ 0.48 and *m*_light_* ≈ 0.41 for the spin-up state and *m*_heavy_* ≈ 0.35 and *m*_light_* ≈ 0.21 for the spin-down state), indicating that the electronic band degeneracy plays an insignificant role in the transport properties of the material.

### Electronic Transport Properties

**Figure 5:**
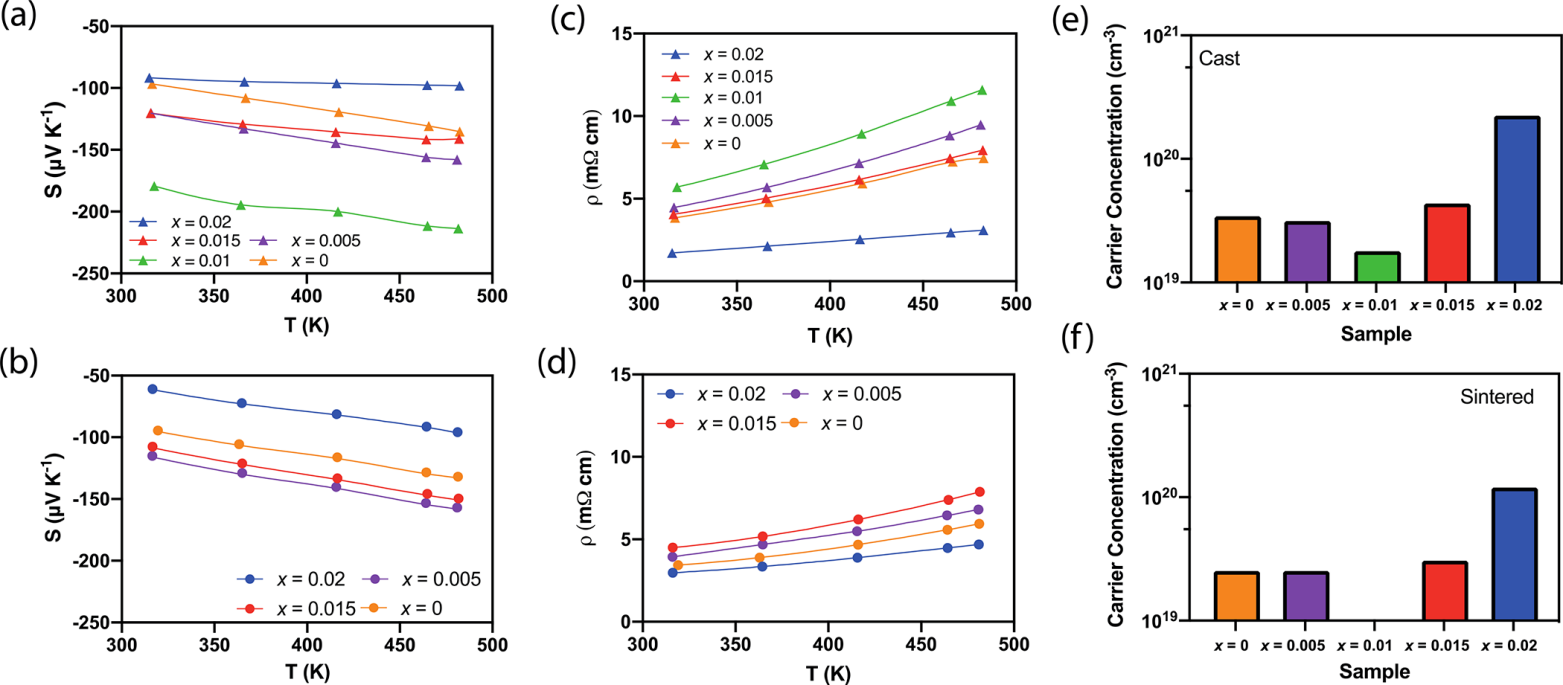
(a, b) Seebeck coefficients, (c, d) electrical resistivities, and (e, f) Hall carrier concentrations of cast Bi_2–*x*/3_Cr_*x*/3_S_3–*x*_Cl_*x*_ (*x* = 0.00, 0.005, 0.01, 0.015, 0.02) and sintered Bi_2–*x*/3_Cr_*x*/3_S_3–*x*_Cl_*x*_ (*x* = 0.00, 0.005, 0.015, 0.02), parallel to the direction of sintering as a function of temperature, respectively.

The Seebeck coefficient, the electrical resistivity, and the carrier concentration of the cast samples of Bi_2–*x*/3_Cr_*x*/3_S_3–*x*_Cl_*x*_ (*x* = 0.00, 0.005, 0.01, 0.015, 0.02) and sintered samples of Bi_2–*x*/3_Cr_*x*/3_S_3–*x*_Cl_*x*_ (*x* = 0.00, 0.005, 0.015, 0.02) measured parallel to the direction of sintering are presented in Figure 5. The negative Seebeck coefficient indicates an n-type semiconductor behavior (Figure 5a,b). The Seebeck coefficient for the cast pristine Bi_2_S_3_ sample ranges from −96 μV K^–1^ at ∼320 K to −135 μV K^–1^ at ∼480 K. These values are considerably smaller than the reported values of −380 to 498 μV K^–1^ for Bi_2_S_3_ in the literature.^26,38^ Following Mott’s formula for the Seebeck coefficient,^51^, the sharp decrease in the Seebeck coefficient can be explained by an increase in the charge carrier density in the material. This is supported by the electrical resistivity values for these samples, which varied from 3.16 mΩ cm at ∼320 K to −4.82 mΩ cm at ∼480 K (Figure 5c). These values, including for *x* = 0, are significantly smaller than the reported values of ∼2400^41^ and ∼7460 mΩ cm^52^ for the pristine sample of Bi_2_S_3_. These results can be explained by the volatile nature of sulfur during the sample fabrication. A single sulfur atom vacancy donates two free electrons to the bulk material. Atom vacancies in bismuth sulfide have been previously reported,^37,38^ and they commonly occur in chalcogenides.^53,54^ This is supported by the high charge carrier concentrations measured for both cast and sintered samples (Figure 5e,f). This also greatly reduces the resistivity for the heavily doped samples, reaching 4.82 mΩ cm at ∼480 K for *x* = 0.02 in comparison to 7.46 mΩ cm for the pristine sample at room temperature. No significant difference was observed in the Seebeck coefficient values of sintered samples for both measurement directions. However, the electrical resistivity of the samples parallel to the direction of sintering is slightly lower than those perpendicular to the sintering direction (Figure S3 in the Supporting Information). The Seebeck coefficient values of sintered samples are very similar to the values obtained from ingots (Figure 5a,b), except for the Seebeck coefficient of the sample with *x* = 0.02, for which the Seebeck coefficient decreased from ∼−100 to ∼−60 μV K^–1^. Overall, the electrical resistivities of the sintered samples are lower than those of their cast counterparts. This is attributed to the improved mechanical integrity of sintered samples relative to the cast samples. The sintered samples with *x* = 0.015, 0.02 showed a smaller reduction in resistivity in comparoson to those with *x* = 0, 0.005, due to the changes in the sintering conditions, which caused the former samples to be less dense than the latter (the sintering temperature was reduced from 723 to 623 K for the samples with *x* = 0.015, 0.02). The reproducibility of the results was verified by repeating the experiments several times (shown in Figure S5 in the Supporting Information).

**Figure 6:**
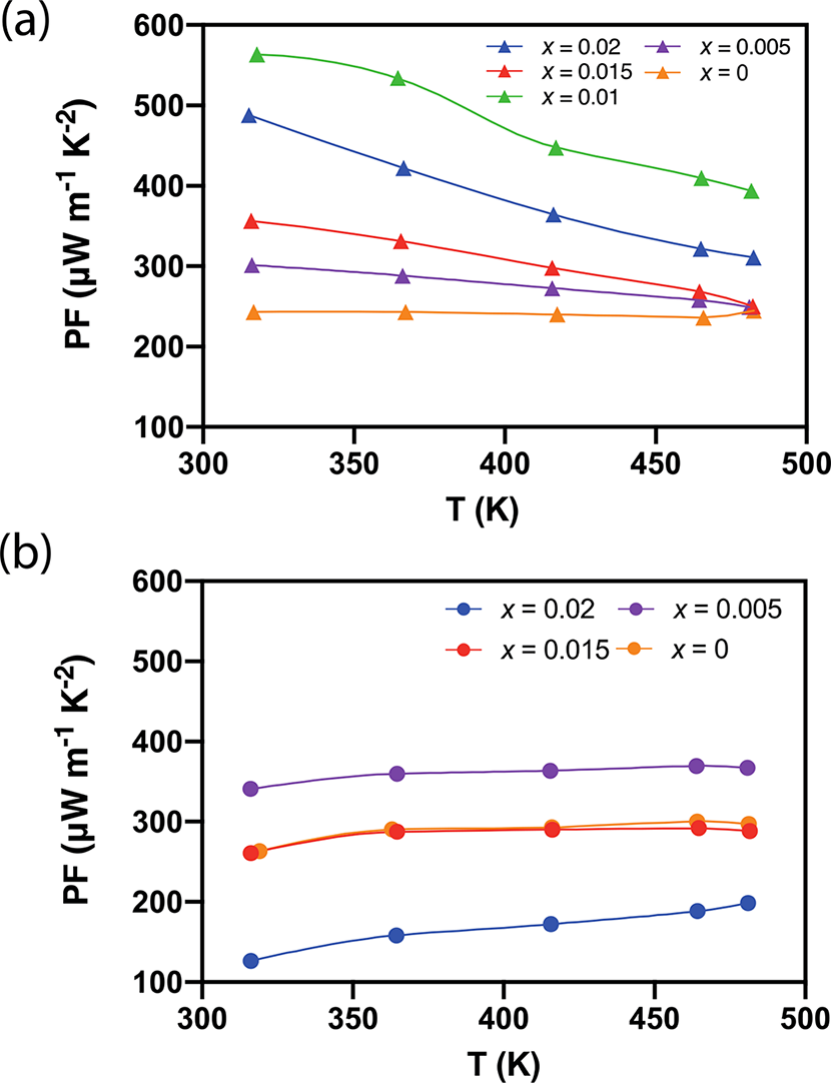
Power factors of (a) cast Bi_2–*x*/3_Cr_*x*/3_S_3–*x*_Cl_*x*_ (*x* = 0.00, 0.005, 0.01, 0.015, 0.02) and of (b) sintered Bi_2–*x*/3_Cr_*x*/3_S_3–*x*_Cl_*x*_ (*x* = 0.00, 0.005, 0.015, 0.02) along the parallel direction of the sintering pressure as a function of temperature.

The power factors (PFs; *S*^2^/ρ) of the cast and sintered samples were measured parallel to the direction of sintering (Figure 6). The PF values of the doped samples are much higher than those of the pristine samples due to the optimization of the electrical conductivity and Seebeck coefficient. The cast Bi_2_S_3_ sample with moderate doping (*x* = 0.01) exhibited the highest PF value (∼566 μW m^–1^ K^–2^ at 320 K), which was about 2.3 times higher than that of the undoped Bi_2_S_3_ sample (about 243 μW m^–1^ K^–2^ at 320 K). However, the sintered sample with *x* = 0.01 was unavailable for measurement. The highest power factor for the sintered sample (*x* = 0.005, measured along the parallel direction to the sintering pressure) was ∼367 μW m^–1^ K^–2^ at 480 K (Figure 6b).

**Figure 7:**
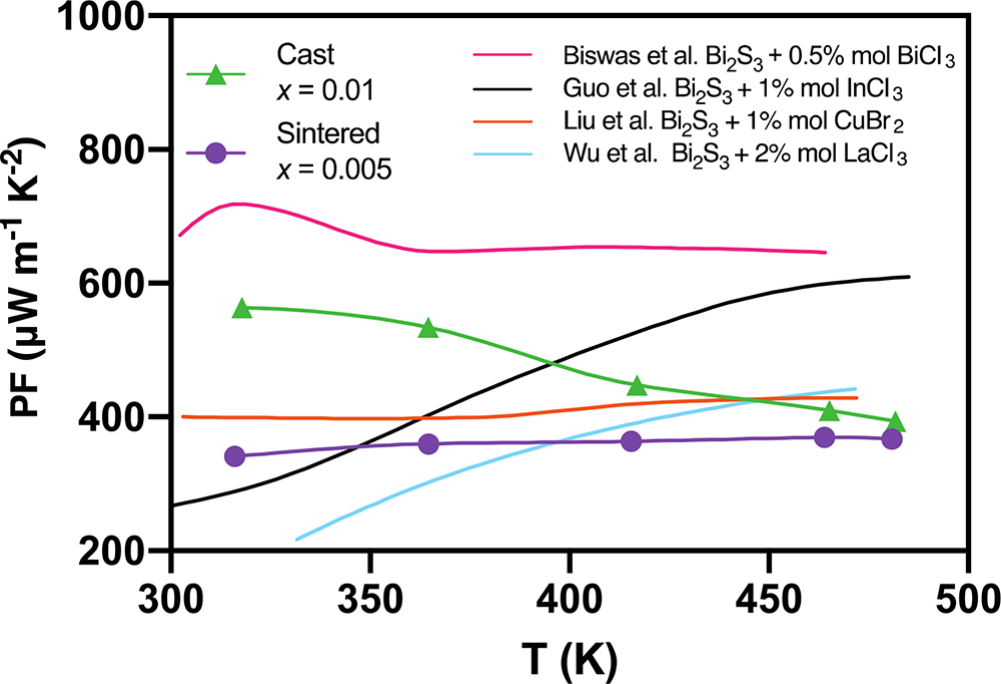
Power factor comparison of n-type Bi_2_S_3_ doped with 0.5% mol of BiCl_3_,^36^ 2% mol of InCl_3_,^35^ 2% of LaCl_3_,^50^ and 1% of CuBr_2_^28^ with sintered Bi_2–*x*/3_Cr_*x*/3_S_3–*x*_Cl_*x*_ (*x* = 0.005) and cast Bi_2–*x*/3_Cr_*x*/3_S_3–*x*_Cl_*x*_ (*x* = 0.01) as a function of temperature.

The PFs obtained in this work are compared with the data reported in the literature (Figure 7). Our results are comparable with the highest values reported in the literature at the same temperature.

Since the samples in the current study have been codoped with Cr and Cl, the relation between the measured Seebeck coefficient and carrier concentration from the cast samples are compared with those of previous studies of Bi_2_S_3_ doped with BiCl_3_,^36^ InCl_3_,^35^ LaCl_3_,^50^ CuBr_2_,^28^ and Cl,^55^ to illustrate the effect of doping with chromium^56^ (Figure 7). The effective mass was evaluated using the single parabolic band (SPB) model with acoustic phonon scattering.^57^ The model uses a Fermi integral of^58,59^
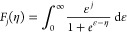
1where η = *E*_F_/(*k*_B_*T*) is the reduced Fermi level and ε is the reduced energy of the electron state. The Seebeck coefficient and the carrier concentration are given by
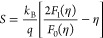
2
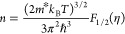
3where m* is the effective mass.

**Figure 8:**
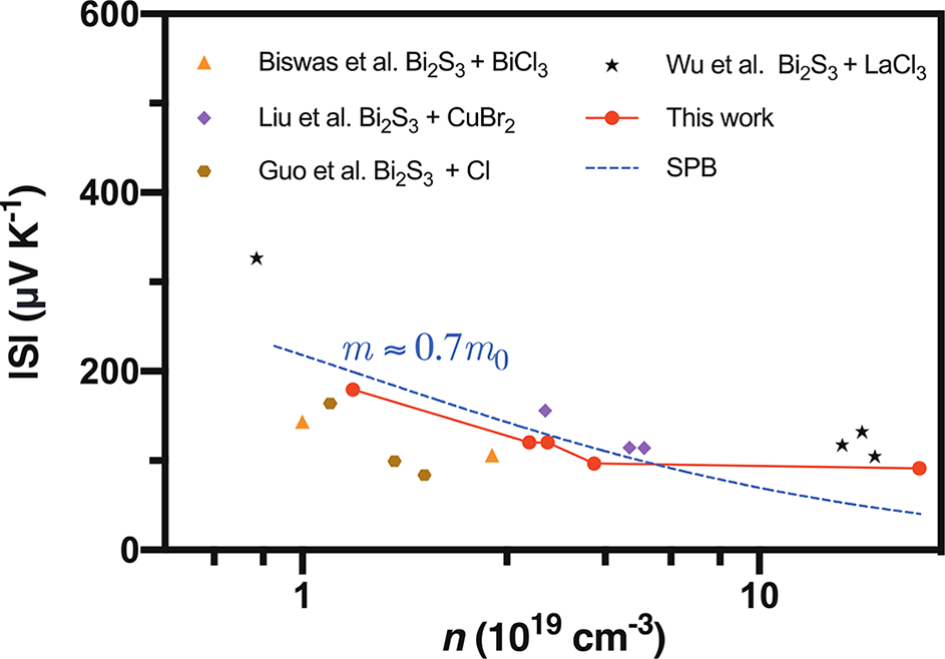
Hall carrier concentration dependence on the room-temperature Seebeck coefficient of n-type cast Bi_2–*x*/3_Cr_*x*/3_S_3–*x*_Cl_*x*_ compared to those reported in the literature of Bi_2_S_3_ doped with BiCl_3_,^13^ LaCl_3_,^35^ CuBr_2_,^5^ and Cl.^40^

For degenerate semiconductors, according to the Pisarenko relation,^60^ the Seebeck coefficient is inversely proportional to the carrier concentration, *n*, with a dependence of *n*^–2/3^. The experimental data of this study deviates from this ideal relationship, which indicates the changes in the electronic band structure of the material.^61^ In particular, the Seebeck coefficient values of the current study are higher than values predicted by the SPB model and experimental data of samples doped only with Cl^35,36^ (as seen in Figure 8). An increase in the Seebeck at a particular carrier concentration was observed in samples doped with La^35^ (due to the presence of La nanoprecipitates) and CuBr_2_ (due to the energy filtering effect^62^). It is worth noting that although Cu is not a magnetic element, it interacts with magnets.

The higher values of the Seebeck coefficient obtained in the current study might be attributed to a magnetic drag effect generated by the magnetic chromium dopant.^18−23^ It has been shown, for example, in the case of magnetic materials that an additional contribution to the Seebeck coefficient is observed when the materials are subjected to a temperature gradient, due to the flux of magnons.^63,64^ The interaction between magnons and carriers results in an overall increase in the effective mass and, consequently, in the Seebeck coefficient.^65^ Similar Seebeck enhancement effects have been observed for nonmagnetic materials doped with magnetic elements, similarly to the present case.^18,19,21,24^ In the present study, the effective mass of the cast samples increased significantly from 0.7*m*_0_ for the pristine sample to 2.1*m*_0_ for the sample with *x* = 0.02 (Table 1), where *m*_0_ is the electron rest mass. This enhanced mass contributed to the higher Seebeck coefficient in comparison with materials doped only with Cl,^36,55^ and it supports the hypothesis of carrier interactions with magnetic elements. The carrier mobilities also decreased with an increase in the concentration of chromium (Table 1). The reduction of charge carrier mobility is responsible for a decrease in the electrical conductivity.^66,67^ However, the overall effect was an increase in the power factor for the lightly doped sample, given the enhanced Seebeck coefficient due to the increased effective mass.

**Table 1:** Carrier Concentration, Mobility, and Calculated Effective Mass of Cast Bi_2–*x*/3_Cr_*x*/3_S_3–*x*_Cl_*x*_

sample (Bi_2–*x*/3_Cr_*x*/3_S_3–*x*_Cl_*x*_)	*n* (10^19^ cm^–3^)	μ (cm^2^ V^–1^ s^–1^)	m*/*m*_0_
*x* = 0	3.44	15.1	0.76
*x* = 0.005	3.14	28.2	0.79
*x* = 0.01	1.79	24.5	0.83
*x* = 0.015	4.35	16.7	0.75
*x* = 0.02	22.4	7.59	2.10

For the sintered samples, the measured carrier concentrations were 2.54 × 10^19^, 2.56 × 10^19^, 3.08 × 10^19^, and 1.2 × 10^20^ cm^–3^ and the mobilities were 60.4, 47.8, 40, and 53.3 cm^2^ V^–1^ s^–1^ for sintered Bi_2–*x*/3_Cr_*x*/3_S_3–*x*_Cl_*x*_ (*x* = 0.00, 0.005, 0.015, 0.02), respectively.

**Figure 9:**
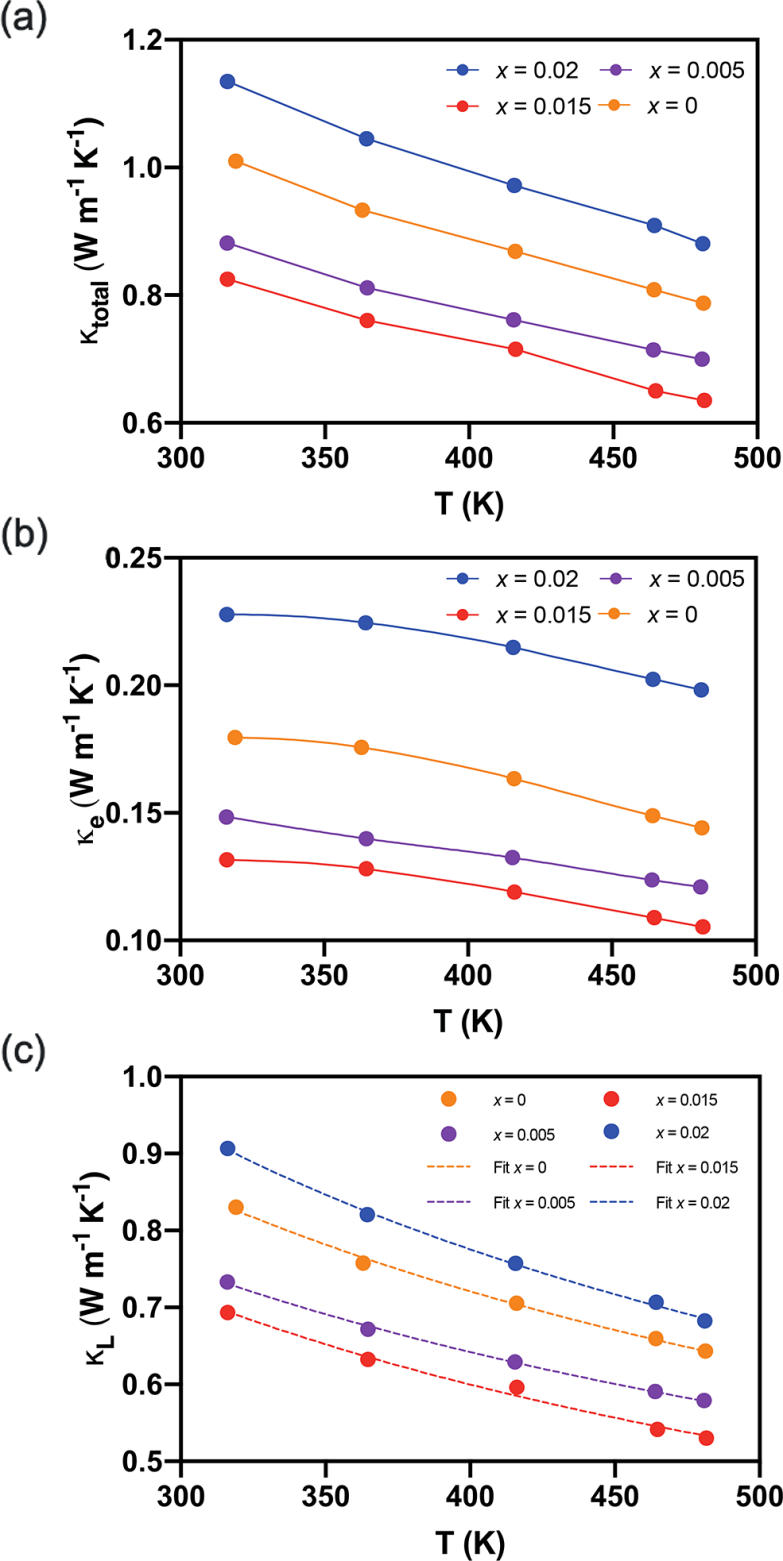
(a) Total thermal conductivity, (b) electronic thermal conductivity; (c) and lattice thermal conductivity (the dashed lines are the calculations based on the Debye–Callaway model) of sintered Bi_2–*x*/3_Cr_*x*/3_S_3–*x*_Cl_*x*_ (*x* = 0.00, 0.005, 0.015, 0.02) parallel to the direction of sintering as a function of temperature.

The temperature dependences of κ_total_, κ_e_ and κ_L_ for sintered Bi_2–*x*/3_Cr_*x*/3_S_3–*x*_Cl_*x*_ (*x* = 0.00, 0.005, 0.015, 0.02) samples measured parallel to the direction of sintering are presented in Figure 9. The total thermal conductivity is the sum of the electronic and lattice thermal conductivity κ_L_ = κ_total_ – κ_e_.

The electronic thermal conductivity, κ_e_, was obtained using the Wiedemann–Franz law, which is expressed as κ_e_ = *L*σ*T*. The Lorenz number (*L*) values as a function of temperature were estimated from the SPB model (Figure S4 in the Supporting Information):^57^
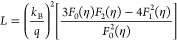
4The values of the electronic thermal conductivity (Figure 9b) are larger for the doped samples. given their higher carrier concentrations (Figure 5f). The values of the lattice thermal conductivity for all samples are very close to the values of *κ*_total_ (Figure 8a,c), due to a small contribution of electronic thermal conductivity to the total thermal conductivity of Bi_2_S_3_.

The κ_total_ values of all the samples ranged from ∼0.8 to ∼1.1 W m^–1^ K^–1^ at 320 K and ranged from ∼0.6 to ∼0.8 W m^–1^ K^–1^ at 480 K (Figure 9a). The samples that were sintered at the lower temperature of 673 K (*x* = 0.015, 0.02) have greater thermal conductivity. Nevertheless, all samples have similar values of lattice thermal conductivity (Figure 9c). The reproducibility of the thermal diffusivity results was verified by repeating the experiment several times; the results are shown in the Figure S6 in the Supporting Information.

To further study this and the effect of the dopant on the scattering mechanism of phonons in these samples, the Debye–Callaway model was adopted to evaluate the thermal conductivity^68,69^
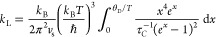
5where *x* = *ℏ*ω/*k*_B_*T* is the reduced frequency, ω the phonon angular frequency, *k*_B_ the Boltzmann constant, *v*_s_ the speed of sound, *ℏ* the reduced Planck constant, *θ*_D_ the Debye temperature, and *τ*_C_ the combined phonon relaxation time. The values of θ_D_ = 283 K and *v*_s_ = 2775 m s^–1^ were adopted from the literature.^70^

Four mechanisms of phonon scattering were considered: point impurities, a normal three-phonon process, an Umklapp process, and boundary scattering.^71^ Matthiessen’s rule^72^ is employed to find the combined phonon relaxation time
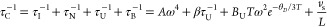
6where τ_I_, τ_N_, τ_U_, and τ_B_ are respectively the relaxation times for points impurity scattering, a normal three-phonon process, an Umklapp process, and boundary scattering, *L* is the average grain size, and the coefficients *A*, β, and *B*_U_ are fitting parameters. Table 2 presents the calculated parameters for all sintered samples parallel to the direction of sintering. The average grain size was obtained from the Rietveld refinement of XRD patterns obtained from samples. The fitted values are shown by dashed lines in Figure 9c.

**Table 2:** Calculated Parameters for the Debye–Callaway Model for Sintered Bi_2–*x*/3_Cr_*x*/3_S_3–*x*_Cl_*x*_ (*x* = 0.00, 0.005, 0.015, 0.02) Samples Parallel to the Direction of Sintering

*x*	*A* (10^–41^ s^3^)	β	*B*_U_ (10^–18^ s K)	*L* (μm)
0	4.9	2.2	3.6	1.3
0.005	7.3	6.3	1.4	1.4
0.015	5.6	6.4	2.0	1.3
0.02	3.5	2.4	3.9	1.5

The results show a noticeable increase in the scattering by point defects with increasing dopant concentration. In general, the thermal conductivity values of the sintered samples are similar for all samples. The changes in β and *B*_U_ indicate that the main mechanism causing these differences was due to changes in the phonon–phonon scattering.

**Figure 10:**
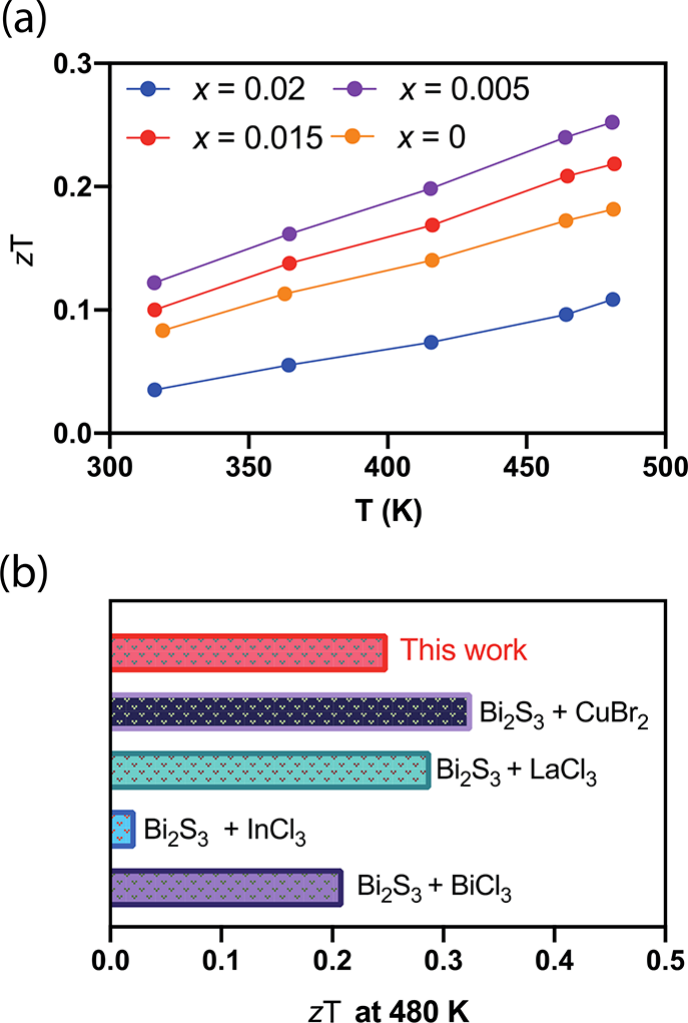
(a) *zT* values of sintered Bi_2–*x*/3_Cr_*x*/3_S_3–*x*_Cl_*x*_ (*x* = 0.00, 0.005, 0.015, 0.02) parallel to the direction of sintering as a function of temperature. (b) *zT* values of sintered Bi_2–*x*/3_Cr_*x*/3_S_3–*x*_Cl_*x*_ (*x* = 0.005) at 480 K in comparison to those of BiCl_3_,^36^ 2 mol % of InCl_3_,^35^ 2 mol % of LaCl_3_,^50^ and 1 mol % of CuBr_2_.^28^

Figure 10 shows the *zT* values for the sintered samples (measured parallel to the direction of sintering). The maximum *zT* value of ∼0.25 was achieved for the sample with *x* = 0.005 at 480 K. It is worth noting that the sample Bi_2–*x*/3_Cr_*x*/3_S_3–*x*_Cl_*x*_ (*x* = 0.01) with the potentially highest *zT* value was unavailable in the sintered form for measurement. Figure 10b compares the *zT* values of the samples in the current study samples with the largest values reported in the literature at the same temperature. There is a difference in the *zT* values obtained from measurements performed parallel and perpendicular to the direction of sintering, due to the crystal structure of Bi_2_S_3_ (Figure S3 in the Supporting Information).

## Conclusions

Bi_2_S_3_ was successfully doped with CrCl_3_ using a melting–annealing technique followed by sintering by the SPS. The electronic properties were measured for both the cast and sintered samples. In comparison to samples with nonmagnetic dopants, the Seebeck coefficient increased at the same carrier concentration, which was most likely due to the magnon drag effect, where the interaction between magnons and carriers effectively increases the effective mass of the carriers and consequently the Seebeck coefficient. The increase in the effective mass led to a decrease in the carrier mobility and the electrical conductivity of the samples with higher carrier concentration. Thermal conductivity measurements of the sintered samples showed similar values for all the samples, with differences arising from the carrier concentration and increased scattering due to impurities. The *zT* values of this work are comparable to the largest values reported in literature and provided experimental evidence that the presence of magnetic dopants can increase the overall efficiency of thermoelectric materials.

## Supporting Information Available

Measured density of the samples, powder diffraction patterns and refined lattice parameters of sintered samples, transport properties and figure of merit of samples, perpendicular to the direction of sintering, heat capacity and Lorenz number used in the thermal conductivity calculations, additional measurements for the transport properties, and error analysis for the Seebeck coefficient and resistivity (PDF)
