# Supplementary material for: Thermoelectric Performance of n-Type Magnetic Element Doped Bi2S3
Source: ACS Appl Energy Mater. 2022 Mar 1;5(3):3845–53. doi: 10.1021/acsaem.2c00295 (PMC9096796; doi:10.1021/acsaem.2c00295)
Supplement: Supplementary file 1 — ae2c00295_si_001.pdf [file ae2c00295_si_001.pdf]

Thermoelectric performance of *n*-type magnetic element doped Bi_2S_3

Raphael Fortulan[a], Sima Aminorroaya Yamini[a,b]*, Chibuzor Nwanebu[a], Suwei Li[c], Takahiro Baba[d,e], Michael John Reece[c], Takao Mori[d,e]

[a] Materials and Engineering Research Institute, Sheffield Hallam University, Sheffield S1 1WB, UK
[b] Department of Engineering and Mathematics, Sheffield Hallam University, Sheffield S1 1 WB, UK
[c] School of Engineering and Material Science, Queen Mary University of London, Mile End Road, London, E1 4NS, UK
[d] International Center for Materials Nanoarchitectonics (WPI-MANA), National Institute for Materials Science, Tsukuba, 305-0044, Japan
[e] Graduate School of Pure and Applied Science, University of Tsukuba, Tsukuba, 305-8577, Japan

*Email: S.Aminorroaya@shu.ac.uk

Table S1. The density of Cast Samples of $Bi_{2-x/3}Cr_{x/3}S_{3-x}Cl_x$ (x = 0.00, 0.005, 0.01, 0.015, 0.02)

Sample ($Bi_{2-x/3}Cr_{x/3}S_{3-x}Cl_x$)	Density (g/cm^{-3})
x = 0	6.59
x = 0.005	6.55
x = 0.01	6.65
x = 0.015	6.45
x = 0.02	6.68

Table S2. The density of Sintered Samples of $Bi_{2-x/3}Cr_{x/3}S_{3-x}Cl_x$ (x = 0.00, 0.005, 0.015, 0.02)

Sample ($Bi_{2-x/3}Cr_{x/3}S_{3-x}Cl_x$)	Density (g/cm^{-3})
x = 0	6.65
x = 0.005	6.70
x = 0.015	6.48
x = 0.02	6.63

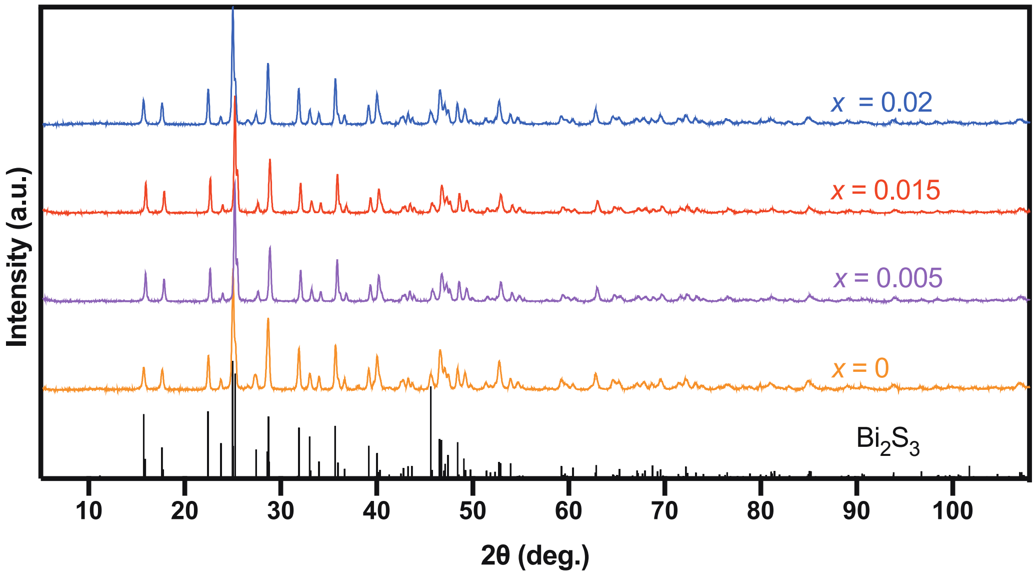

Figure S1. Powder X-Ray Diffraction Patterns of Sintered $Bi_{2-x/3}Cr_{x/3}S_{3-x}Cl_x$ (x = 0.00, 0.005, 0.015, 0.02) samples in the range of 5° to 108°

Table S3. Rietveld refined lattice parameters of cast $Bi_{2-x/3}Cr_{x/3}S_{3-x}Cl_x$ (x = 0.00, 0.005, 0.01, 0.015, 0.02) samples

Sample ($Bi_{2-x/3}Cr_{x/3}S_{3-x}Cl_x$)	a (Å)	b (Å)	c (Å)
x = 0	$11.29 \pm 8\times10^{-5}$	$3.98 \pm 1\times10^{-5}$	$11.14 \pm 1\times10^{-4}$
x = 0.005	$11.29 \pm 2\times10^{-4}$	$3.99 \pm 8\times10^{-5}$	$11.14 \pm 3\times10^{-4}$
x = 0.01	$11.28 \pm 5\times10^{-4}$	$3.99 \pm 3\times10^{-5}$	$11.14 \pm 5\times10^{-4}$
x = 0.015	$11.29 \pm 2\times10^{-4}$	$3.98 \pm 1\times10^{-4}$	$11.14 \pm 3\times10^{-4}$
x = 0.02	$11.29 \pm 2\times10^{-4}$	$3.98 \pm 2\times10^{-4}$	$11.14 \pm 4\times10^{-4}$

Table S4. Rietveld refined lattice parameters of sintered $Bi_{2-x/3}Cr_{x/3}S_{3-x}Cl_x$ (x = 0.00, 0.005, 0.015, 0.02) samples

Sample ($Bi_{2-x/3}Cr_{x/3}S_{3-x}Cl_x$)	a (Å)	b (Å)	c (Å)
x = 0	$11.29 \pm 2.8\times10^{-4}$	$3.98 \pm 1.2\times10^{-4}$	$11.14 \pm 3.9\times10^{-4}$
x = 0.005	$11.29 \pm 1.8\times10^{-4}$	$3.98 \pm 8.7\times10^{-5}$	$11.14 \pm 2.5\times10^{-4}$
x = 0.015	$11.28 \pm 1.7\times10^{-4}$	$3.98 \pm 8.3\times10^{-5}$	$11.14 \pm 2.4\times10^{-4}$
x = 0.02	$11.29 \pm 2.2\times10^{-4}$	$3.98 \pm 1.0\times10^{-4}$	$11.14 \pm 4.5\times10^{-4}$

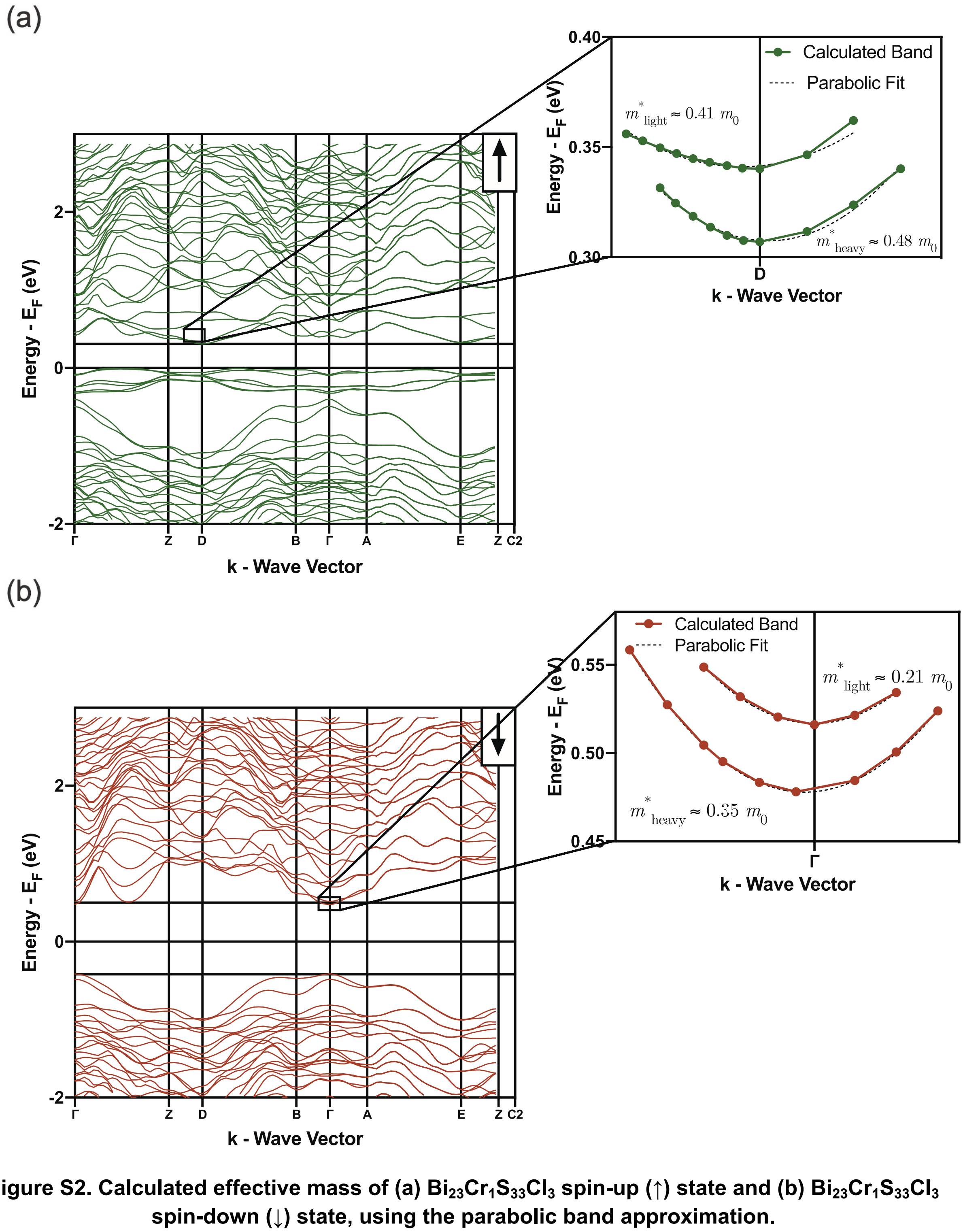

Figure S2. Calculated effective mass of (a) $Bi_{23}Cr_1S_{33}Cl_3$ spin-up (↑) state and (b) $Bi_{23}Cr_1S_{33}Cl_3$ spin-down (↓) state, using the parabolic band approximation.

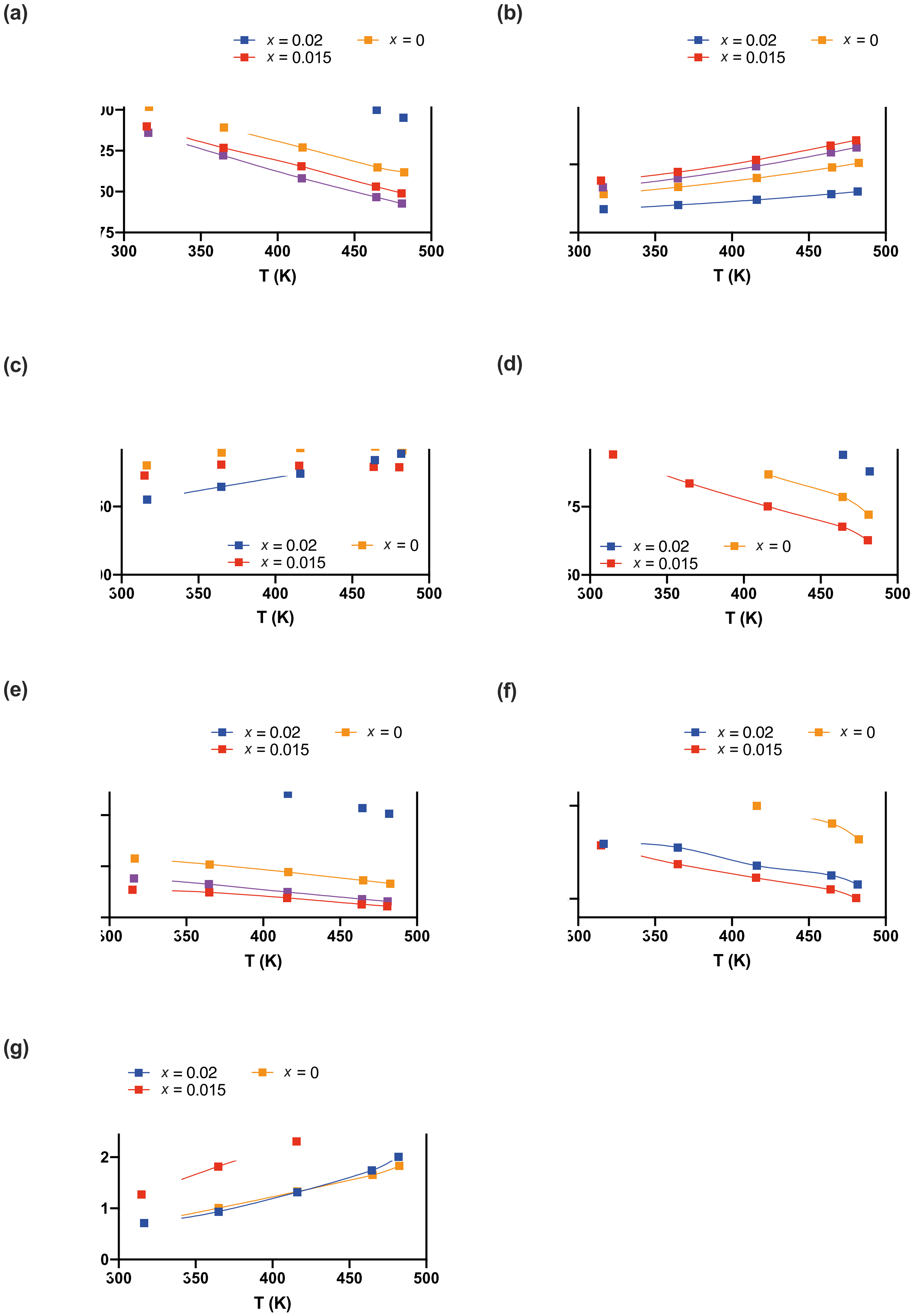

Figure S3 – (a) The Seebeck coefficient; (b) the electrical resistivity; (c) the power factor; (d) the total thermal conductivity; (e) the electronic thermal conductivity; (f) the lattice thermal conductivity; and (g) *z*T of sintered $Bi_{2-x/3}Cr_{x/3}S_{3-x}Cl_x$ (*x* = 0.00, 0.005, 0.015, 0.02) parallel to the direction of sintering as a function of temperature.

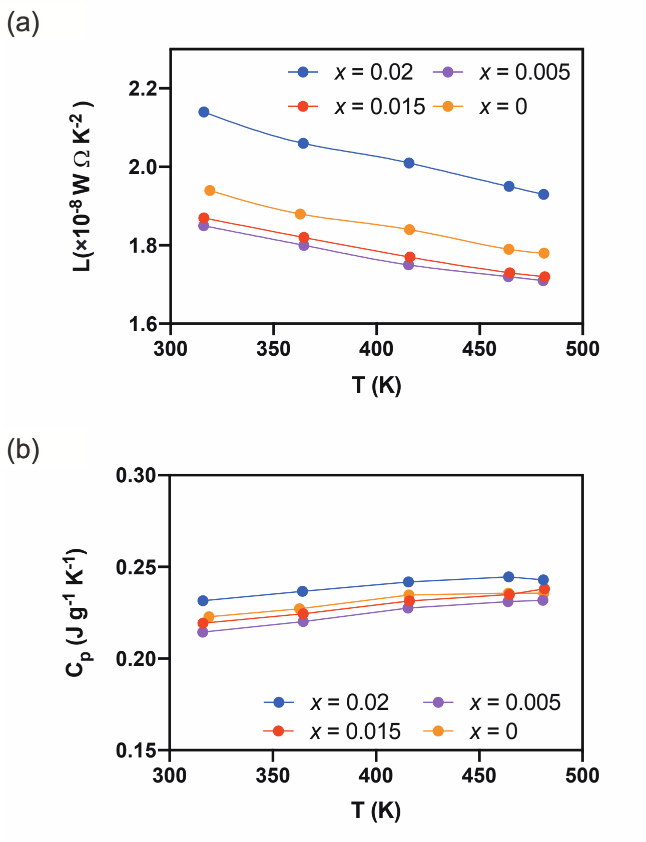

Figure S4. (a) The Lorenz number and (b) the Heat capacity of sintered $Bi_{2-x/3}Cr_{x/3}S_{3-x}Cl_x$ (x = 0.00, 0.005, 0.015, 0.02) parallel to the direction of sintering as a function of temperature, respectively.

Error Analysis for Seebeck coefficient and resistivity measurements

To estimate the error in our measurements, we followed the bootstrap approach[1] using Monte Carlo resampling (1×10^4 samples). The sources of error considered for the analysis were:

1) Resistivity: I) Probe spacing (±0.1 mm), II) Bar dimensions (±0.01 mm), III) Voltage (±5%), and III) Current (0.2% + 0.3 mA)[2];
2) Seebeck Coefficient: I) Cold-finger effect[2], II) Voltage (±5%), III) Absolute Temperature (±2 K), and IV) Estimate standard error from the slope approach method.

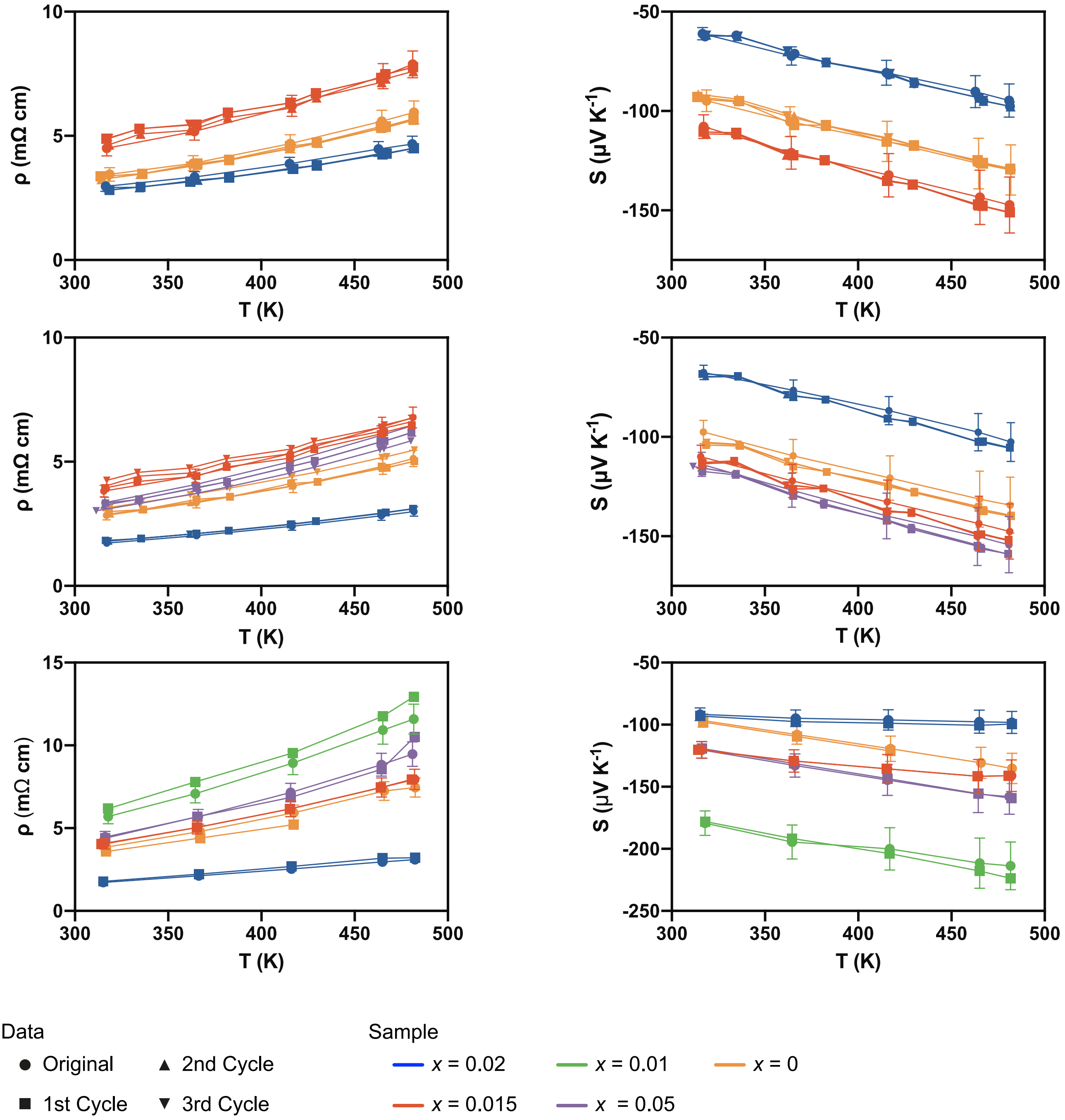

Figure S5. Cyclic measurements and errors bars for (a) the Seebeck coefficient, (b) the electrical resistivity of sintered $Bi_{2\text{-}x/3}Cr_{x/3}S_{3\text{-}x}Cl_x$ (x = 0.00, 0.005, 0.015, 0.02)perpendicular to the direction of sintering as a function of temperature; (c) The Seebeck coefficient, (d) the electrical resistivity of sintered $Bi_{2\text{-}x/3}Cr_{x/3}S_{3\text{-}x}Cl_x$ (x = 0.00, 0.005, 0.015, 0.02) parallel to the direction of sintering as a function of temperature; (e) The Seebeck coefficient, (f) the electrical resistivity of cast $Bi_{2\text{-}x/3}Cr_{x/3}S_{3\text{-}x}Cl_x$ (x = 0.00, 0.005, 0.01,0.015, 0.02) as a function of temperature.

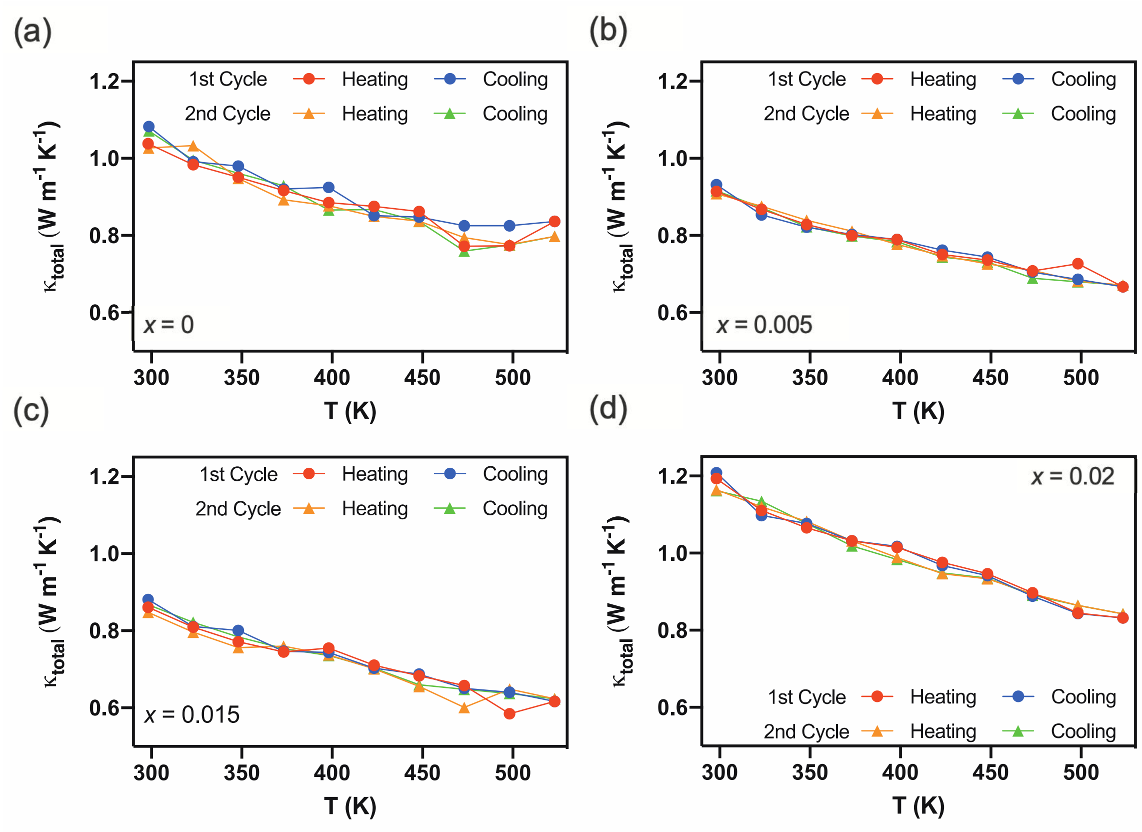

Figure S6. Cyclic measurements for the thermal conductivity of $Bi_{2-x/3}Cr_{x/3}S_{3-x}Cl_x$ for (a) x = 0, (b) x = 0.005, (b) x = 0.015, and (d) x = 0.02 parallel to the direction of sintering as a function of temperature.

REFERENCES

(1) Bradley Efron; Robert J. Tibshirani. *An Introduction to the Bootstrap*; Monographs on Statistics and Applied Probability; Chapman & Hall/CRC: Boca Raton, Florida, USA, 1993.

(2) Mackey, J.; Dynys, F.; Sehirlioglu, A. Uncertainty Analysis for Common Seebeck and Electrical Resistivity Measurement Systems. *Review of Scientific Instruments* **2014**, *85* (8), 085119. https://doi.org/10.1063/1.4893652.
